# Leveling the Playing Field: A New Proposed Method to Address Relative Age- and Maturity-Related Bias in UK Male Academy Soccer Players

**DOI:** 10.3389/fspor.2022.847438

**Published:** 2022-03-03

**Authors:** Sofie Bolckmans, Janet L. Starkes, Chris Towlson, Chris Barnes, Guy Parkin, Werner F. Helsen

**Affiliations:** ^1^Department of Movement Sciences, KU Leuven, Leuven, Belgium; ^2^Institute of Exercise Training and Sport Informatics, German Sport University Cologne, Köln, Germany; ^3^Department of Kinesiology, McMaster University, Hamilton, ON, Canada; ^4^Department of Sport, Health and Exercise Science, University of Hull, Hull, United Kingdom; ^5^CB Sports Performance Ltd., Rugeley, United Kingdom; ^6^Staffordshire University, Stoke on Trent, United Kingdom; ^7^Pro Football Support, Huddersfield, United Kingdom

**Keywords:** relative age effect, maturity status, developmental birthdate, allocation date, talent identification

## Abstract

Relative age selection bias persists within all major soccer leagues and youth soccer academies across the globe, with the relative age effect (RAE) being typically characterized as the over selection of relatively older players (who have sometimes also been shown to be early maturing). The aim of this study was to examine if a new allocation method (i) eliminates the RAE, and (ii) reduces the presence of any additional maturity-related differences in anthropometric and physical fitness characteristics which may exist between players within the same selection category. In the first phase, 1,003 academy soccer players [under (U) 9–16] from 23 UK professional soccer clubs were sampled and a clear RAE per birth quarter (Q) was observed for the overall sample (Q1 = 45.0% vs. Q4 = 9.8%) as well as for the different age categories. Using the newly suggested reallocation method, youth players were divided by allocation date which was defined as the midway point between the chronological age and the estimated developmental (ED) birthdate. Stature was used as an anthropometric reference point on the P50 of the growth curve to determine the developmental birthdate for this new method. After the reallocation of the players using ED, the distribution of players was more equally spread (Q1 = 25.3%, Q2 = 25.6%, Q3 = 22.4%, Q4 = 26.7%). After reallocation, the mean delta stature was reduced by 16.6 cm (from 40.3 ± 7.08 to 23.7 ± 4.09 cm, d = 2.87). The mean delta body mass difference after allocation was reduced by 6.7kg (from 33.2 ± 6.39 to 26.5 ± 4.85 kg, d = 1.18). The mean age difference increased from 1.8 to 3.9 years. A total of 42.7% of the sample would have been reallocated to a different age group compared to the current one. After reallocation, 45% of the anthropometric and physical fitness comparisons showed reductions in the within-group variation expressed as a percentage of coefficient of variation (CV%). The U10 players demonstrated the largest reduction in CV% (−7.6%) of the anthropometric characteristics. The U10 squad also showed the largest reduction in CV% for various physical fitness characteristics (−7.5%). By both eliminating the RAE and reducing temporary maturity-related anthropometric and physical fitness differences, soccer academies across the world may diversify and increase the size of the talent pool both for clubs and national youth teams. In conclusion, this study provides further evidence that the newly proposed allocation method shows the potential to remove the RAE and to create a more “leveled playing field” by reducing the within-group variation of anthropometric and physical fitness characteristics affording relatively younger, and eventually, late-maturing players the opportunity to develop their talent fairly.

## Introduction

Academy soccer players' [e.g., under (U)9 to U23] development and talent identification are multifaceted and complex (Larkin and O'Connor, 2017; Towlson et al., 2019, 2021a; Doncaster et al., 2020; Romann, 2020). Both require soccer practitioners (e.g., coaches, sports scientists, performance analysts, etc.) to make informed judgements relating to the players' physical, technical, tactical, and psycho-social characteristics (Larkin and O'Connor, 2017; Towlson et al., 2019). Historically, relative age (i.e., players birthdate in relation to the cut-off dates for the domestic soccer season; Helsen et al., 1998, 2000, 2005), and maturity-related selection bias (Lovell et al., 2015; Towlson et al., 2017) onset by the highly individualized timing of the adolescent growth spurt (Philippaerts et al., 2006) [typically reported as estimated (Fransen et al., 2021) peak height velocity (PHV) (Mirwald et al., 2002; Moore et al., 2015; Fransen et al., 2018; Kozieł and Malina, 2018) or percentage of final adult stature (Khamis and Roche, 1994; Towlson et al., 2021c)] have been offered as separate (Helsen et al., 2005) and collective (Cobley et al., 2009; Lovell et al., 2015) causal factors for the overrepresentation of players who are either relatively older and/or early maturing. Both methods are commonly utilized by professional soccer academies (Salter et al., 2021). Such selection biases likely lead to homogenous pools of players selected for academy soccer programs who are often either relatively older and/or who are early maturing in comparison to population norms (Lovell et al., 2015; Towlson et al., 2017). These early maturing players are often characterized as possessing temporary, maturity-related enhancements in anthropometric (e.g., stature and body mass) and physical fitness characteristics (e.g., power, strength, speed) (Towlson et al., 2018, 2021c). Such temporary advanced somatic characteristics are often perceived important by talent practitioners (Towlson et al., 2019), characterize key tactical roles (Deprez et al., 2015; Towlson et al., 2017), and can influence passing patterns during match-play, with later maturing players becoming subconsciously over-reliant on their early maturing counterparts. Subsequently, this can lead to a (sub)conscious reduction in selection opportunities for relatively younger players, who may also be less mature (i.e., smaller and less physically developed), but who are of equally technical and tactical abilities (Abbott et al., 2019). Recent studies (Brustio et al., 2018) have highlighted that the birthdate distribution of young professional youth soccer players clearly showed a relative age effect. This trend was maintained, to a lesser extent, in senior elite teams, for example in the Italian professional soccer league *Serie* A. Therefore, the relatively older individuals have more chances to be selected by elite teams, both in young and senior categories. In fact, this selection bias limits the possibility to potentially select talented athletes born late in the year of consideration. At the senior professional level, a large RAE is evident in all popular Italian team sports, however, soccer is the most affected sport (Lupo et al., 2019). However, although we acknowledge previous works which have shown relatively older players to be early maturing (Helsen et al., 2012), recent academy-based soccer research (Johnson et al., 2017; Parr et al., 2020; Towlson et al., 2021b) suggests that relative age and maturity-related selection bias should be considered as separate entities (Towlson et al., 2021b). For instance, Hill et al. (2020) provided evidence to suggest that relative age and maturity selection bias can both confound academy soccer talent selection and development strategies. However, the timing of both these effects is asynchronous, with relative age bias having a stronger presence from late childhood (age 6–7 years) and across adolescence. In contrast, maturity selection bias, in the form of early maturing male soccer players likely being selected based on their enhanced anthropometric and physical fitness characteristics, emerge during puberty alone. In turn, this observation suggests that relative age and maturity selection bias are independent challenges for academy soccer practitioners which may confound their efforts to accurately identify talented soccer players. Therefore, there is a need for new and innovative methods which can tackle both, despite their individualized timing of effect.

Multiple solutions have been offered by researchers to alleviate the separate selection biases onset by both relative age (Helsen et al., 2005, 2012; Cobley et al., 2009; Mann and van Ginneken, 2017; Kelly et al., 2020) and timing of maturation (Cumming et al., 2017; Malina et al., 2019). Several proposals have been suggested to remove relative age bias including changing (Barnsley, 1988; Helsen et al., 2012) and rotating the cut-off dates (Barnsley, 1988), installing sport-specific cut-off dates (Musch and Grondin, 2001), and particular [i.e., age-ordered numbered soccer shirts and birthday banding (Kelly et al., 2020)] talent identification strategies (Mann and van Ginneken, 2017). Maturity-related selection bias between players' differences has been addressed using maturity bio-banding (Reeves et al., 2018; Abbott et al., 2019; Macmaster, 2021; Towlson, 2021; Towlson et al., 2021a), whereby players are grouped according to their maturation status (Cumming et al., 2017; Malina et al., 2019). Despite the relative success of some of the aforementioned methods to reduce RAE (i.e., birthday and bio-banding) and maturity selection bias (i.e., bio-banding), many proposed solutions can be difficult to implement and rely on the flexibility of coaches in how they structure games and training programs. Consequently, this reallocation method and methods like bio-banding should be used to supplement chronological playing groups to enhance the development of players. In response, Helsen et al. (2021) have developed a new, cost-effective, simple, and practical way (i.e., absence of maturity estimation equations and/or player radiographs) to categorize players using an anthropometric-based (i.e., stature) estimated developmental birthdate (Helsen et al., 2021). To simplify the reallocation for soccer academies, the Developmental Age Calculator (2021) calculates the midway point between the decimal age and the estimated developmental (ED) birthdate. The ED birthdate was estimated by comparing the anthropometric characteristics of each player with the normative growth curves from a longitudinal study examining secular changes in biological maturation in Belgian boys of the same age categories (Roelants et al., 2009). The anthropometric characteristics of each player were plotted on the corresponding 50th percentile curve to determine the player's ED age for stature.

Early evidence (Romann, 2020) suggested that ED birthdate may possess the capacity to remove the RAE in academy elite youth soccer players (arbitrary cut-off dates categories: Q1 = 41.4 vs. Q4 = 14.9%; ED birthdate reallocation Q1 26.5; Q2 21.9; Q3 27.5; Q4 24.2%; *n* = 302). In addition, similar to the bio-banding (Macmaster, 2021) the ED birthdate method has also been shown to reduce player differences in stature (mean difference = 16.6 cm) and body mass (mean difference = 6.7 kg) owing to the individualized timing and effect of maturation (Towlson et al., 2018). However, more research is required to explore the efficacy of this method and establish if the ED birthdate is a viable method to replace existing player categorization methods. Therefore, the aims of this study were to examine if the ED categorization method (i) eliminates the RAE, and (ii) reduces the presence of any additional maturity-related differences in anthropometric and physical fitness characteristics between teammates using a broad sample (*n* = 1,003) of academy soccer players (under 9–16) from 23 different UK professional soccer academies.

## Methods

### Phase I: Regrouping Youth Players Using the Midway Point Between the Chronological and Estimated Developmental Birthdates

#### Participants

Following the Ethics Committee Approval (1415038), a retrospective, cross-sectional, convenience sample of 1,003 male soccer players U18 (U9: *n* = 61, U10: *n* = 111, U11: *n* = 113, U12: *n* = 123, U13: *n* = 106, U14: *n* = 212, U15: *n* = 126, U16: *n* = 26, U17: *n* = 95, U18: *n* = 27) participating in 23 different UK professional soccer academies during the competitive seasons 2011–2012, 2012–2013, and 2013–2014 participated in the study. The youngest player was born on 9 November 2004 (8.3Y) and the oldest player on 25 June 1994 (18.9Y). Data were collected between 4 November 2011 and 30 April 2014. Due to the low statistical power, players within the U9 and U16 (or above) were deselected (*n* = 39) from the initial sample. This was considered justified because of the small relevance of these squads to the effect of RAE (Helsen et al., 2021). Players who were not reallocated by the ED age calculator were deselected because their anthropometric parameters were outside of the limits for normal growth curves. These procedures deselected 191 players from the initial sample of 1,003 players and resulted in a sample of 812 academy soccer players.

#### Procedures

**Figure 1:**
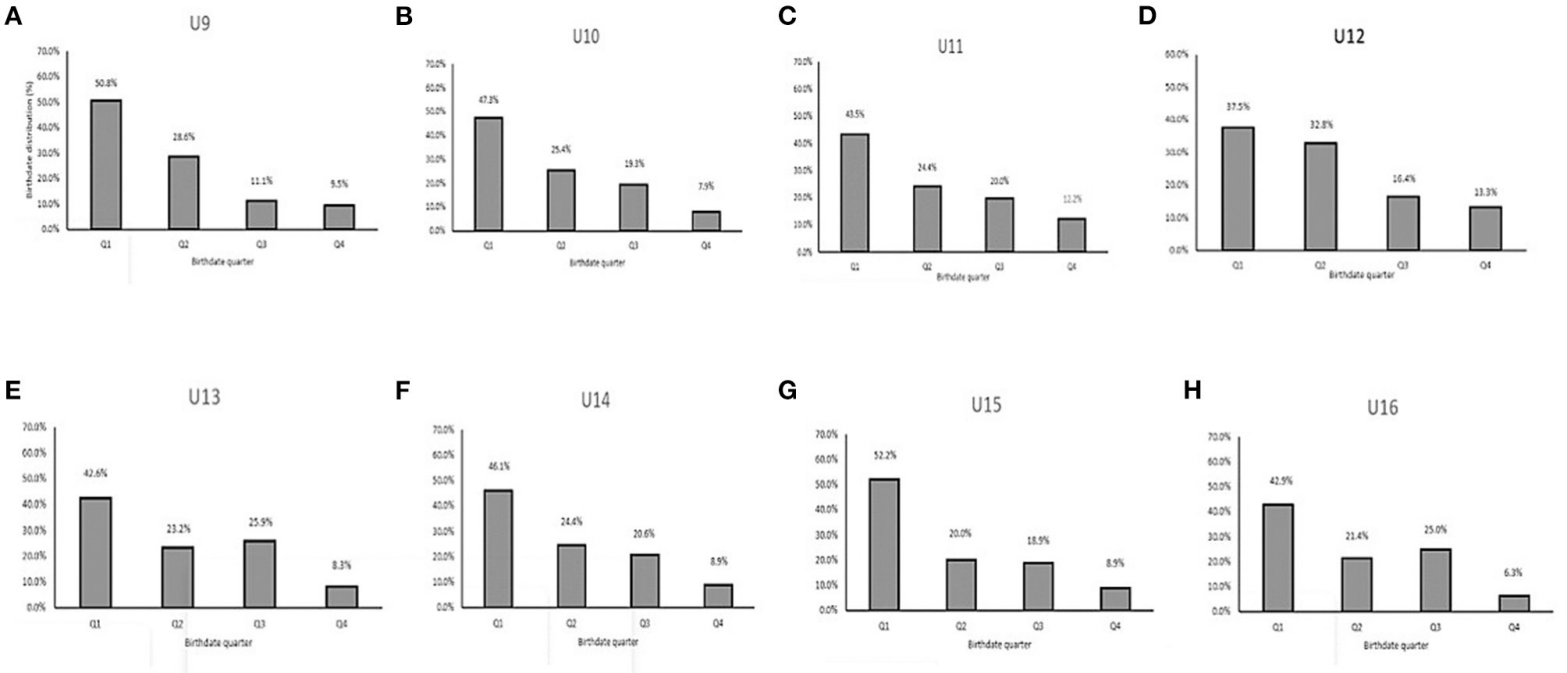
Chronological birthdate distribution (%) of 812 UK youth soccer players per quarter for every age category before reallocation. **(A)** U9. **(B)** U10. **(C)** U11. **(D)** U12. **(E)** U13. **(F)** U14. **(G)** U15. **(H)** U16.

Using previously published methods (Helsen et al., 2005; Steingröver et al., 2017), players were grouped within each category according to typical UK domestic soccer season birth quartiles (Q1: 1 September−30 November; Q2: 1 December−29 February; Q3: 1 March−31 May; Q4 1 June−31 August) and expressed as a percentage of the sample population (Figures 1, 3). The players' dates of birth were attained from club records and categorized into birth quartiles (Q) within each specific age category.

Mean age, stature, and body mass difference (delta) per age category were established chronologically (Table 1) and according to ED birthdate (Table 2). The ED birthdate was estimated by comparing the anthropometric characteristics of each player with the normative growth curves from a longitudinal study examining secular changes in biological maturation in Belgian boys of the same age categories (Roelants et al., 2009).

**Table 1:** Number of players (*n*) by squad with mean, minimal and maximal anthropometric characteristics before reallocation.

**Squad**	***n***	**Mean stature (cm)**	**Min. stature (cm)**	**Max. stature (cm)**	**Mean body mass (kg)**	**Min. body mass (kg)**	**Max. body mass (kg)**	**Mean age (Y)**	**Delta age (Y)**	**Delta body mass (kg)**	**Delta stature (cm)**
U9	63	139.23	122.65	156.6	33.81	24.3	52.6	9.39	1.53	28.3	43.95
U10	114	141.3	126	177.4	35.3	27.0	69.0	10.19	1.82	42.0	51.4
U11	115	144.93	129.9	171.15	37.46	24.5	58.3	11.10	2.22	33.8	41.3
U12	128	151.16	135.1	170.3	40.5	20.2	57.4	12.11	2.02	37.2	35.2
U13	108	158	139.8	179.3	46.8	30.2	68.2	13.10	1.97	38.0	39.5
U14	180	165.42	113.9	180.3	53.22	31.3	68.7	14.08	1.61	37.4	66.4
U15	90	171.13	153.4	181.0	60.15	41.1	69.2	15.43	1.75	28.1	27.6
U16	14	174.07	164.3	181.0	62.61	48.7	69.3	16.19	1.36	20.6	16.7
**Average**									**1.8**	**33.2**	**40.3**

*Delta values indicate variation by squad*.

*Cm, centimeter; Kg, kilogram; Min., minimum; Max, maximum*.

**Table 2:** Number of players (n) by squad with mean, minimal and maximal anthropometric characteristics after reallocation.

**Squad**	***n***	**Mean stature (cm)**	**Min. stature (cm)**	**Max. stature (cm)**	**Mean body mass (kg)**	**Min. body mass (kg)**	**Max. body mass (kg)**	**Mean age (Y)**	**Delta age (Y)**	**Delta body mass (kg)**	**Delta stature (cm)**
U9	57	135.06	126	142.17	31.5	26.0	40.9	9.79	2.35	14.9	16.17
U10	118	139.20	113.9	152.0	33.9	24.5	62.8	10.21	4.98	38.3	38.1
U11	115	145.03	137.9	166.6	37.2	30.1	52.6	11.16	4.40	22.5	28.7
U12	119	150.87	139.8	170.6	40.78	23.7	59.2	12.10	4.14	17.08	30.8
U13	128	159.0	145.0	177.4	47.3	20.2	69.0	12.98	5.61	48.8	32.4
U14	133	164.89	153.3	173.8	52.85	30.6	67.7	14.11	3.42	37.1	20.5
U15	95	171.8	164.3	179.3	59.8	48.7	69.2	14.87	3.73	20.5	15.0
U16	28	176.67	171.4	179.65	63.34	56.2	69.3	15.23	2.57	13.1	8.25
**Average**									**3.9**	**26.5**	**23.7**

*Delta values indicate variation by squad*.

*Cm, centimeter; Kg, kilogram; Min., minimum; Max, maximum*.

#### Anthropometric and Maturation Status Measures

The players' stature and body mass were collected using previously outlined methods (Towlson et al., 2018, 2021a; Helsen et al., 2021). Stature (seca 217, Chino, U.S.A.) and body mass (seca Robusta 813, Chino, USA) were collected by suitably trained members of the coaching staff. All measures were collected in duplicates. If measures differed by >*0.4* cm or >*0.4* kg, a third measure was taken and the median value was recorded (Lovell et al., 2015; Towlson et al., 2018). Leg length was estimated by subtracting seated stature from standing stature. The anthropometric measures were used in conjunction with player age and test date to estimate players' somatic maturity (Lovell et al., 2015; Towlson et al., 2018). Each player's predicted age at peak height velocity (PHV) was estimated using a cross-validated algorithm (Philippaerts et al., 2006; Lovell et al., 2015; Towlson et al., 2018). Table 1 provides an overview of the mean, minimal, and maximal anthropometric characteristics per age category with the corresponding delta values.

#### New Player Allocation Method

The 50th Percentile (P50) of the normative growth curve of the Belgian population (Hauspie et al., 1993) was used to establish the developmental birthdate (Leite Portella et al., 2017). The anthropometric characteristics of each player were plotted on the corresponding 50th percentile curve to determine the player's ED age for stature. For example, if a male player was 132.4-cm tall, his ED age is 8 years and 6 months because 50% of the children are 132.4-cm tall. Stature was used as an anthropometric reference point in a separate established ED age evaluation to determine developmental birthdate on the P50 of the growth curve, though stature and body mass are, in general, highly correlated (Helsen et al., 2021). Moreover, muscle mass can be profoundly influenced by external factors like muscle growth hormones and strength training (Leite Portella et al., 2017). Therefore, only stature was considered a reference point for this new method. Stature cannot be interpreted as a measure of maturation; it has just been used as a way of establishing a biologically anchored birthdate (using normative growth curves) in which to recategorize the child. It was anticipated that the ED birthdate may lead to large age differences in the players' chronological age. Therefore, the allocation date (AD) was determined as the midway point between chronological and developmental birthdates to provide the ED. It was used to allocate the player to a higher or lower age category or maintain the same age category if necessary. For example, a U11 player with a calculated allocation date of 6 March 2002 was allocated to the U10 category. However, with an allocation date of 14 February 2003, the player might be allocated to the U9 squad. The ED birthdate was calculated with a customized spreadsheet (Helsen et al., 2021). This developmental age calculator was developed to estimate the developmental age and allocation age of players, based on normal growth curves. Phase 1 of the study assessed the effectiveness of the new method to (i) eliminate the RAE and (ii) reduce maturity-related differences in anthropometric and physical fitness characteristics between players. Specifically, the number of players who were reallocated to a lower or higher age category or remained in the same age category was quantified. Finally, an analysis was made between the differences (delta) in the selection method with chronological age, developmental age, and the new allocation method, according to decimal age, stature, body mass, and physical parameters (Tables 1–5). The overarching aim of the present study was to assess if the new method of player categorization creates a more “level playing field” by reducing the within group variation of anthropometric and physical parameters which have been associated with relative age (Helsen et al., 2021) and the highly individualized timing of maturation (Towlson et al., 2018).

**Table 3:** Delta values indicate variation for physical parameters as agility, jump performance (CMJ), sprint capacity (10 m/20 m) and endurance capacity (MSFT) before allocation.

**Squad**	**Delta agility (s)**	**Delta CMJ (cm)**	**Delta sprint 10m (s)**	**Delta sprint 20m (s)**	**Delta MAS MSFT (Km/h)**	**Delta Distance MSFT (m)**
U9	3.06	17.0	0.53	0.85	2.90	1,240
U10	3.98	23.9	0.61	1.16	1.30	2,220
U11	3.0	24.1	0.51	0.89	3.30	1,440
U12	2.53	30.9	0.49	0.82	3.10	1,340
U13	2.89	23.2	0.48	0.87	3.30	1,460
U14	3.17	35.3	0.62	0.92	12.3	1,460
U15	2.79	31.0	0.51	0.89	2.80	1,340
U16	1.15	24.3	0.20	0.34	1.45	680

*CMJ, counter movement jump; MAS, maximal aerobic speed; MSFT, multi-stage fitness test; s, seconds; m, meter*.

**Table 4:** Delta values indicate variation for physical parameters as agility, jump performance (CMJ), sprint capacity (10 m/20 m) and endurance capacity (MSFT) after allocation.

**Squad**	**Delta agility (s)**	**Delta CMJ (cm)**	**Delta sprint 10 m (s)**	**Delta sprint 20 m (s)**	**Delta MAS MSFT (km/h)**	**Delta Distance MSFT (m)**
U9	2.83	21.1	0.58	0.87	2.6	1,080
U10	3.29	22.0	0.42	0.85	12.0	1,660
U11	3.18	32.4	0.52	0.98	3.5	1,520
U12	2.54	23.1	0.54	0.76	13.3	1,820
U13	3.18	27.5	0.54	1.09	11.5	1,940
U14	2.84	33.3	0.57	0.88	12.3	1,460
U15	2.84	33.9	0.64	0.96	7.2	1,420
U16	2.08	21.7	0.41	0.62	6.8	920

*CMJ, counter movement jump; MAS, maximal aerobic speed; MSFT, multi-stage fitness test; s, seconds; m, meter*.

**Table 5:** Overview of the category changes that result from the new allocation method.

**Squad**	**3 teams down**	**2 teams down**	**1 team down**	**Same team**	**1 team up**	**2 teams up**	**3 teams up**	**Total (n)**	**Not reallocated (%)**	**Reallocated (%)**
U9	0%	0%	4.8%	55.6%	36.6%	3.2%	0%	63	55.6%	44.4%
U10	0%	0%	18.4%	60.0%	14.9%	3.5%	3.5%	114	60.0%	40.0%
U11	0%	0%	20.9%	60.9%	16.5%	1.7%	0%	115	60.9%	39.1%
U12	0%	1.6%	19.5%	56.3%	22.7%	0%	0%	128	56.3%	43.7%
U13	0%	0.9%	21.3%	61.1%	13.9%	2.8%	0%	108	61.1%	38.9%
U14	0.6%	0.6%	15.0%	56.7%	21.7%	3.3%	2.2%	180	56.7%	43.3%
U15	0%	1.1%	17.8%	53.3%	20.0%	5.6%	2.2%	90	53.3%	46.7%
U16	0%	0%	35.7%	28.6%	21.4%	14.3%	0%	14	28.6%	71.4%
**Total**	**0.1%**	**0.6%**	**17.7%**	**57.3%**	**20.1%**	**3.0%**	**1.2%**	**812**	**57.3%**	**42.7%**

*The UK youth players who are not reallocated, are also presented*.

### Phase II: Evaluation of the Within-Group Variation of Anthropometric and Physical Fitness Characteristics Based on Chronological and Estimated Developmental Birthdates

The players' mean age, stature, and body mass with variations (delta) were established by chronological age category (Table 1) and by reallocation date (Table 2) using the methods as described in phase I. Anthropometric variables from each player were recorded as stated in phase I.

#### Physical Fitness Measures

A battery of field tests was performed to assess discrete components of the players' physical fitness. Given that explosive power, agility, acceleration, speed, and endurance are considered important physical parameters that impact the selection process (Philippaerts et al., 2006), these attributes were measured in turn. The players' sprint ability was assessed using the 20 m sprint test (Rodríguez-Fernández et al., 2019). Players were instructed to cover this distance in the fastest possible time. The 20 m sprint was selected because of its relevance with the demands of soccer match-play (Rodríguez-Fernández et al., 2019). Two phases were taken into consideration for analyses: 0–10 m and 10–20 m split times. Three pairs of digital timing gates (Brower Timing System, Salt Lake City, Utah, USA), set at 0, 10, and 20 m were used to record both split times. The players started their attempts from a standing position behind the first digital timing gates. Three trials were performed by each participant, with a 3-min break of passive recovery between the trials. The best result was recorded for further analysis (Tables 3, 4).

**Figure 2:**
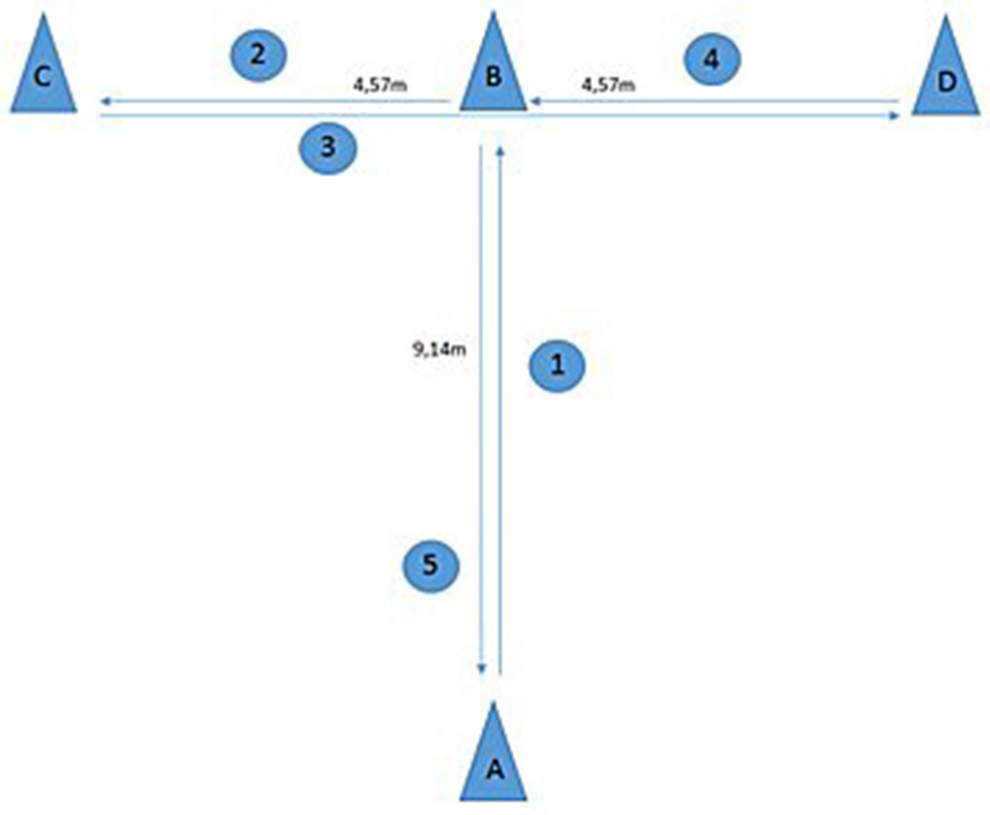
The *T*-Test is a useful agility test for the assessment of multidirectional movement (forward, lateral, and backward). The cones are set up as depicted in the figure. The goal is to complete the course as quickly as possible (A-B-C-D-B-A). The player shuffles through the course between the cones and must physically touch each cone with the correct hand. The fastest time is recorded.

A player's agility was recorded using the methods published for the *T*-test (Negra et al., 2017). The test is a measurement of 4-directional agility and body control that analyses the ability to change directions rapidly while maintaining balance without loss of speed (Negra et al., 2017). Specifically, reference points were placed at 3 cones (B, C, and D) to monitor the consistency and accuracy of the test execution. Players started with both feet behind point A. Each player sprinted forward 9.14 m to point B; then they shuffled to the left 4.57 m to cone C. Participants shuffled then to the right 9.14 m and touched cone (D). Finally, they shuffled to the left 4.57 m back to point B. Players then ran backwards, passing the finishing line at point A. Two test trials were performed, and times were recorded to the nearest one-hundredth of a second using digital timing gates as per previous methods (Negra et al., 2017) (Figure 2; Tables 3, 4).

A player's explosive lower limb power was then assessed using a vertical counter-movement jump (CMJ) (Optojump, Microgate, Bolzano, Italy) according to previously outlined methods (Quagliarella et al., 2011). The player performed two CMJs interspaced by 1 min of passive recovery. If the difference in jump heights differed more than 2 cm, a third jump was recorded (maximum of 8 jumps) with the mean of the highest 3 jumps were recorded (Tables 3, 4) (Towlson et al., 2017; Loturco et al., 2020). Finally, the multi-stage fitness test (MSFT) was used to record aerobic endurance (Lovell et al., 2015). The test involved players running back and forth between two points 20 m apart. Each run was synchronized with a pre-recorded audio track which plays a beep at regular intervals. Over the course of the test, the players advanced through the levels (lasting just over a minute each), with the beeps getting faster at each new level reached. The point where the player fails to reach the 20 m point before the beep was deemed as the player's highest score, and the test ended. Maximal aerobic speed (MAS) and total distance covered was recorded as a surrogate measure of aerobic capacity. The differences (delta) in physical fitness parameters for explosive power, agility, acceleration speed, and endurance per age category were first calculated for chronological date of birth (Table 3). Thereafter, the differences in physical fitness parameters were compared per age category for developmental birthdate and allocation date (Table 4).

### Statistical Analyses

**Figure 3:**
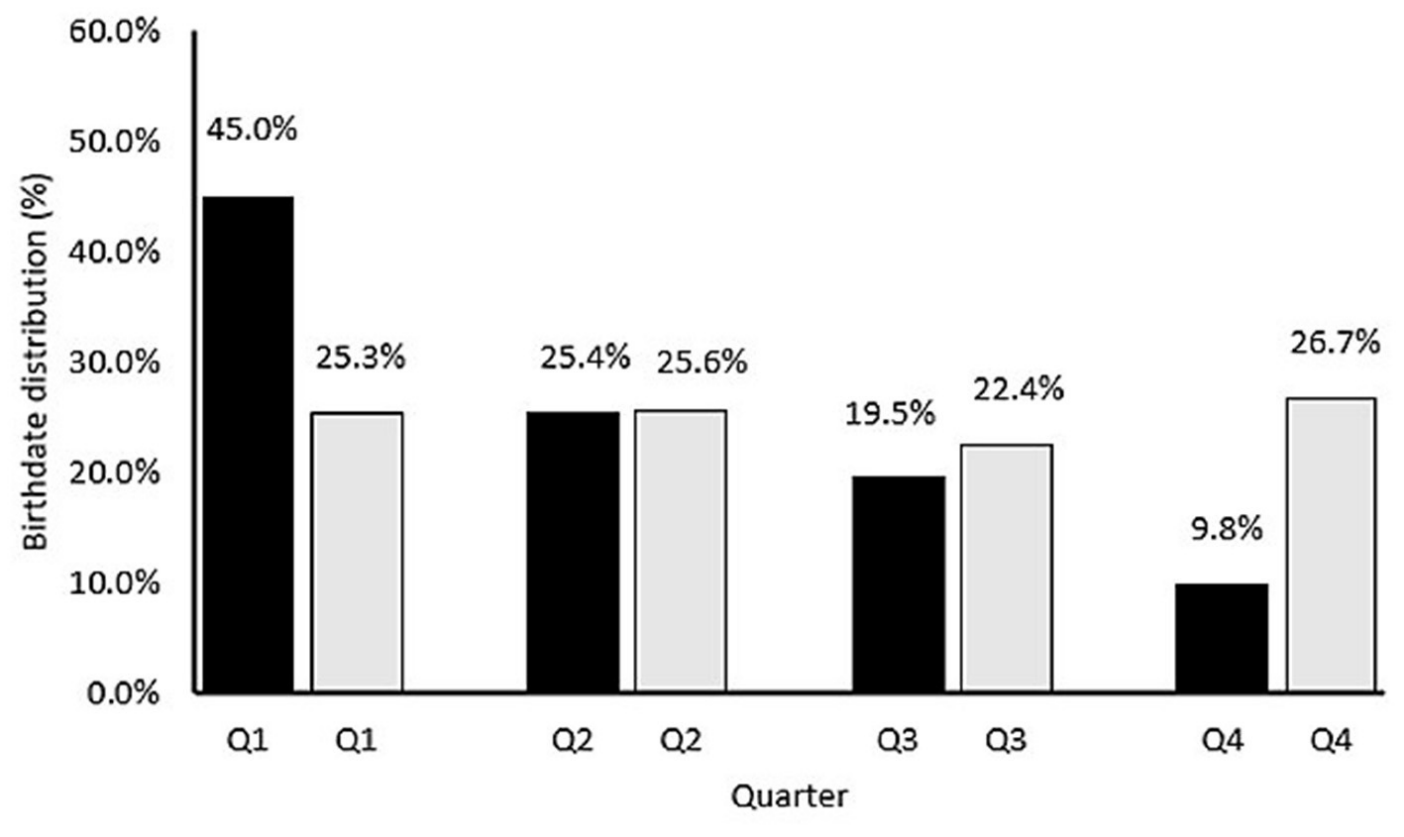
Birthdate distribution (%) of 812 UK academy soccer players by birthdate quarter before (black) and after (gray) reallocation.

For phase I, statistical analyses were performed to examine the relative age effect of the whole data set (Figure 3) and per age category (Figure 1). RAEs were determined using odds ratios (OR). Mean, standard deviation, and median with interquartile range were used to compare numerical data. The effect sizes between categories were calculated with Cohen‘s *d*. Based on Cohen's *d* values, effect sizes can be interpreted as trivial, small, moderate, large, and very large (Table 6) (Fritz et al., 2012). For phase II, the within-group variation of anthropometric and physical fitness parameters for chronological, developmental, and reallocation methods was calculated as the percentage coefficient of variation (CV%). The CV% of different parameters was recorded as the standard deviation of the between the trial difference of each method, divided by the mean between trial difference (Table 6). For the chronological, developmental, and reallocation group, data analysis was presented as mean and Cohen's *d* values for small, medium, and large effects (Fritz et al., 2012). Cohen's *d* (Fritz et al., 2012; Bosco et al., 2015) values were assessed for chronological and reallocation groups with categories: trivial (<0.20), small (0.21–0.60), moderate (0.61–1.20), large (1.21–2.00) and very large (>2.01) (Table 6).

**Table 6:** Summary table of mean ± SD and effect sizes for anthropometrical and physical characteristics of UK academy soccer players (U9–U16) according to traditional chronologically ordered and the proposed reallocation method.

**Banding method**	**Chronological**	**Reallocation**	**Effect size**	**Chronological**	**Reallocation**	**Effect size**	**Chronological**	**Reallocation**	**Effect size**	**Chronological**	**Reallocation**	**Effect size**
**Squad**	**U9**	**U9**	**U9**	**U10**	**U10**	**U10**	**U11**	**U11**	**U11**	**U12**	**U12**	**U12**
**Metrics**												
Stature (cm)	139.2 ± 6.0	135.1 ± 3.5	0.9 (mod.)	141.3 ± 9.6	139.2 ± 4.5	0.3 (small)	144.9 ± 6.9	145.0 ± 4.1	0.02 (trivial)	151.2 ± 6.3	150.9 ± 5.1	0.1 (trivial)
CV %	4.3	2.6		6.8	3.2		4.8	2.8		4.2	3.4	
Body-mass (Kg)	33.8 ± 4.5	31.5 ± 2.9	0.6 (small)	35.3 ± 7.5	33.9 ± 4.6	0.2 (trivial)	37.46 ± 6.2	37.2 ± 4.3	0.05 (trivial)	40.5 ± 6.3	40.78 ± 5.1	0.05(trivial)
CV%	13.3	9.2		21.2	13.6		16.6	11.6		15.6	12.5	
CMJ (cm)	21 ± 4.1	21.3 ± 4.7	0.1 (trivial)	22.4 ± 4.6	23 ± 4.8	0.1 (trivial)	24.9 ± 5.2	24.1 ± 5.5	0.1 (trivial)	24.9 ± 6.1	25.3 ± 5.7	0.1 (trivial)
CV %	19.5	22.1		20.1	20.1		20.9	22.8		24.5	22.5	
Agility (s)	12.08 ± 0.7	11.9 ± 0.6	0.3 (small)	11.53 ± 0.7	11.68 ± 0.6	0.23 (small)	11.32 ± 0.6	11.31 ± 0.6	0.02 (trivial)	11.01 ± 0.5	10.92 ± 0.5	0.2 (trivial)
CV%	5.8	5.0		6.1	5.1		5.3	5.3		4.5	4.6	
Sprint 10 m (s)	1.98 ± 0.1	1.96 ± 0.1	0.2 (trivial)	1.93 ± 0.1	1.96 ± 0.1	0.3 (small)	1.9 ± 0.09	1.9 ± 0.09	0.0 (trivial)	1.86 ± 0.1	1.85 ± 0.1	0.1 (trivial)
CV %	5.1	5.1		5.2	5.1		4.7	4.7		5.4	5.4	
Sprint 20 m (s)	3.61 ± 0.2	3.58 ± 0.2	0.2 (trivial)	3.54 ± 0.2	3.57 ± 0.2	0.2 (trivial)	3.47 ± 0.2	3.47 ± 0.2	0.0 (trivial)	3.39 ± 0.2	3.37 ± 0.2	0.1 (trivial)
CV %	5.5	5.6		5.6	5.6		5.8	5.8		5.9	5.9	
Distance MSFT (m)	1084 ± 259.1	1145 ± 312.7	0.2 (trivial)	1385 ± 446.7	1288 ± 329.0	0.3 (small)	1371.5 ± 291.3	1393.0 ± 295.0	0.1 (trivial)	1508 ± 269.2	1555.6 ± 344.3	0.2 (trivial)
CV %	27.2	27.3		32.2	25.6		27.2	27.3		17.9	22.1	
MAS MSFT (Km/h)	11.7 ± 0.7	11.9 ± 0.7	0.3 (small)	12.65 ± 2.3	12.2 ± 1.3	0.24 (small)	12.3 ± 0.7	12.3 ± 0.7	0.0 (trivial)	12.6 ± 0.6	12.96 ± 1.8	0.3 (small)
CV %	6.0	5.9		18.2	10.7		5.7	5.7		4.8	13.9	
**Squad**	**U13**	**U13**	**U13**	**U14**	**U14**	**U14**	**U15**	**U15**	**U15**	**U16**	**U16**	**U16**
**Metrics**												
Stature (cm)	158 ± 8.2	159.0 ± 5.8	0.1 (trivial)	165.4 ± 8.5	164.9 ± 4.2	0.1 (trivial)	171.1 ± 6.0	171.8 ± 3.3	0.1 (trivial)	174.1 ± 5.1	176.7 ± 2.2	0.8 (mod.)
CV %	5.2	3.6		5.1	2.5		3.5	1.9		2.9	1.2	
Weight (kg)	46.8 ± 7.3	47.3 ± 6.9	0.1 (trivial)	53.2 ± 7.5	52.9 ± 6.3	0.04 (trivial)	60.2 ± 6.0	59.8 ± 5.1	0.1 (trivial)	62.6 ± 5.8	63.3 ± 3.6	0.2 (trivial)
CV%	15.6	14.6		14.1	11.9		10.0	8.5		9.3	5.7	
CMJ (cm)	26.6 ± 5.6	25.9 ± 6.3	0.1 (trivial)	28.5 ± 7.0	28.4 ± 6.9	0.01 (trivial)	29.0 ± 8.3	29.6 ± 7.9	0.1 (trivial)	27.9 ± 7.3	28.1 ± 6.9	0.03 (trivial)
CV %	21.1	24.3		24.6	24.3		28.6	26.7		26.2	24.6	
Agility (s)	10.45 ± 0.5	10.5 ± 0.6	0.1 (trivial)	10.16 ± 0.6	10.14 ± 0.6	0.03 (trivial)	9.7 ± 0.5	9.9 ± 0.6	0.4 (small)	9.5 ± 0.4	9.6 ± 0.5	0.2 (trivial)
CV%	4.8	5.7		5.9	5.9		5.2	6.1		4.2	5.2	
Sprint 10 m (s)	1.78 ± 0.1	1.78 ± 0.1	0.0 (trivial)	1.72 ± 0.1	1.72 ± 0.1	0.0 (trivial)	1.63 ± 0.1	1.65 ± 0.1	0.21 (small)	1.6 ± 0.1	1.6 ± 0.1	0.4 (small)
CV %	5.6	5.6		5.8	5.8		6.1	6.1		6.3	6.1	
Sprint 20 m (s)	3.22 ± 0.2	3.24 ± 0.2	0.1 (trivial)	3.11 ± 0.2	3.11 ± 0.2	0.0 (trivial)	2.94 ± 0.1	2.97 ± 0.2	0.2 (trivial)	2.9 ± 0.1	2.9 ± 0.2	0.0 (trivial)
CV %	6.2	6.2		6.4	6.4		3.4	6.7		3.4	6.9	
Distance MSFT (m)	1,720 ± 305.2	1,685.0 ± 335.2	0.1 (trivial)	1,898 ± 287.7	1,928 ± 287.7	0.1 (trivial)	2,102.3 ± 297.5	2,012.6 ± 329.5	0.3 (small)	2,168.6 ± 203.6	2,035.7 ± 234.3	0.6 (small)
CV %	17.8	19.9		15.2	14.9		14.2	16.4		9.4	11.5	
MAS MSFT (km/h)	13.2 ± 0.7	13.3 ± 1.5	0.1 (trivial)	14.0 ± 1.8	13.9 ± 1.6	0.1 (trivial)	13.96 ± 0.6	14.0 ± 1.2	0.04 (trivial)	14.1 ± 0.4	14.1 ± 1.2	0.0 (trivial)
CV %	5.3	11.3		12.9	11.5		4.3	8.6		2.8	8.5	

*CV%, percentage of coefficient of variation; CMJ, counter-movement jump; MSFT, multi-stage fitness test; U, under; effect size, Cohen's d thresholds were set as trivial (<0.20), small (0.21–0.60), moderate (0.61–1.20), large (1.21–2.00) and very large (>2.01)*.

## Results

### Phase I: Reallocating Youth Players Using the Midway Point Between the Chronological and Estimated Developmental Birthdates

#### Relative Age Effect

The RAE was significant for the overall dataset before reallocation (Figure 3). Players that were born in Q1 had the highest representation (45%), followed by players born in Q2 (25.4%), Q3 (19.5%), and Q4 (9.8%). The RAE was also clearly present in all age categories (U9–U16) (Figure 1). The magnitude of the RAE was a factor of “five” (OR 4.57) for the overall sample of youth players (Q1 = 45 vs. Q4 = 9.8%) (Figure 3). An uneven distribution was identified for each annual group, with 37.5–52.2 % of players born in Q1 and 6.3–13.3% in Q4 (Figure 1). Odds ratios (ORs) were used to estimate the magnitude of the RAE calculated for the whole database and each age category: 4.57 (database), 5.34 (U9), 6 (U10), 3.57 (U11), 2.82 (U12), 5.11 (U13), 5.19 (U14), 5.87 (U15), 6.86 (U16). Table 1 shows the anthropometric parameters according to the chronological age categorized teams with the mean values and variation for each (Delta). The differences in stature (cm) between the tallest and smallest players ranged from 16.7 (U16) to 51.4 (U10). The mean difference in stature across the chronological age groupings was 40.3 cm. The mean difference in body mass across the chronological age was 33.2 kg and the mean difference in age was 1.8 years (Table 1).

#### Effect of Reallocation

**Figure 4:**
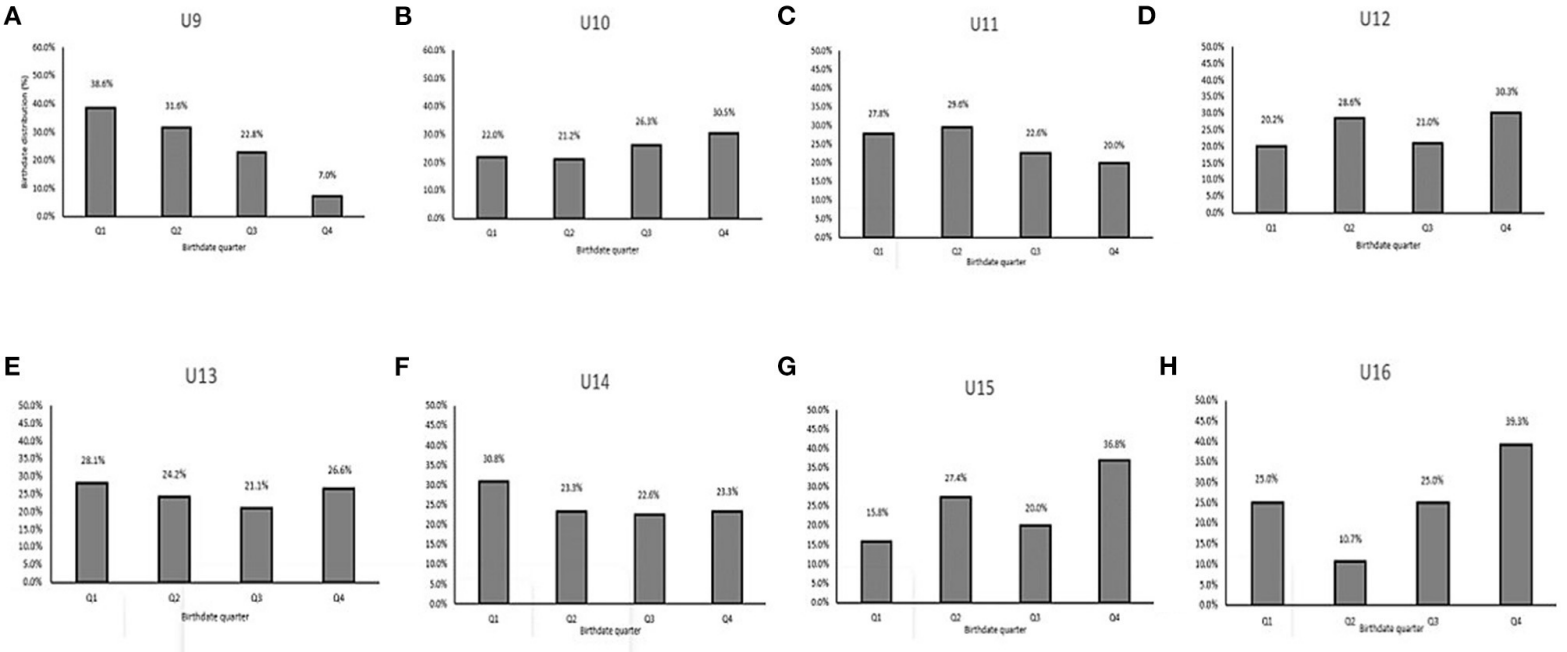
Birthdate distribution (%) of 812 UK youth soccer players by quarter for every age category after reallocation. **(A)** U9. **(B)** U10. **(C)** U11. **(D)** U12. **(E)** U13. **(F)** U14. **(G)** U15. **(H)** U16.

After reallocating the players, the whole-group differences in proportion per birthdate quarter reduced, as shown in Figures 3, 4. Each quarter equated to ~25% of the players after allocating (Q1 = 25.3, Q2 = 25.6, Q3 = 22.4, and Q4 = 26.7%). This pattern was evident in all age categories except for U9 (Figure 4).

**Figure 5:**
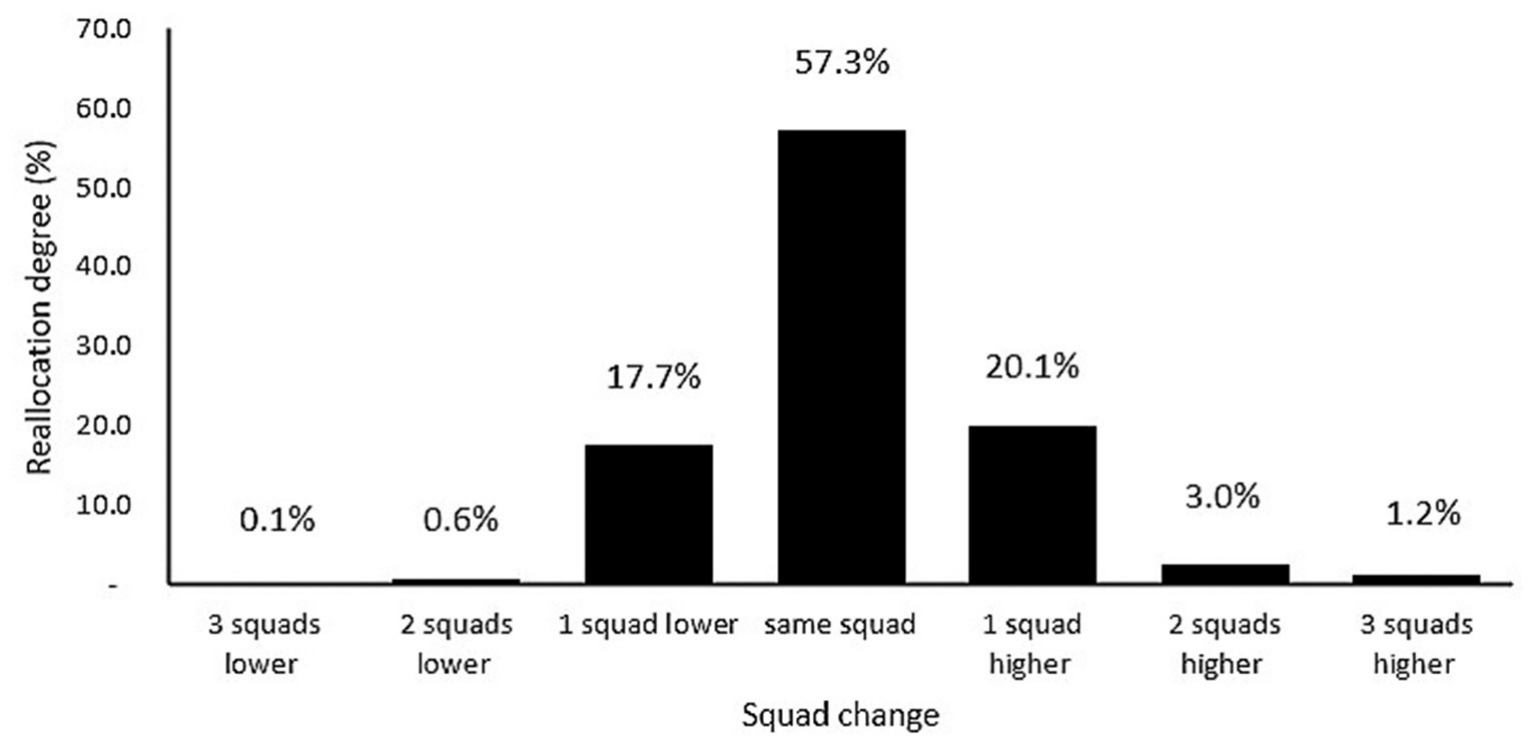
Percentage of players (%) who will be reallocated to another age category.

In Table 2, delta values for age, body mass, and stature are demonstrated for each age category. After reallocation, delta age values increase and delta body mass and stature values decrease for each age category. After allocation, mean delta age values for all groups increase (1.8 vs. 3.9 years), whereas mean delta stature (40.3 vs. 23.7 cm) and delta body mass (33.2 vs. 26.5 kg) values decrease (Table 2). In Table 5 and Figure 5, squad changes are listed for each age category after allocation. The reallocation method resulted in 42.7% of players moving age-groups with 17.7% one age-group lower, 20.1% one age-group higher, 3% two age-groups higher, and finally 1.2% three age categories higher (Figure 5; Table 5). Further, 0.1% was reallocated three age categories lower and 0.6% two age categories lower (Figure 5; Table 5).

### Phase II: Evaluation of the Within-Group Variation of Anthropometric and Physical Fitness Characteristics Based on Chronological and Estimated Developmental Birthdates

**Figure 6:**
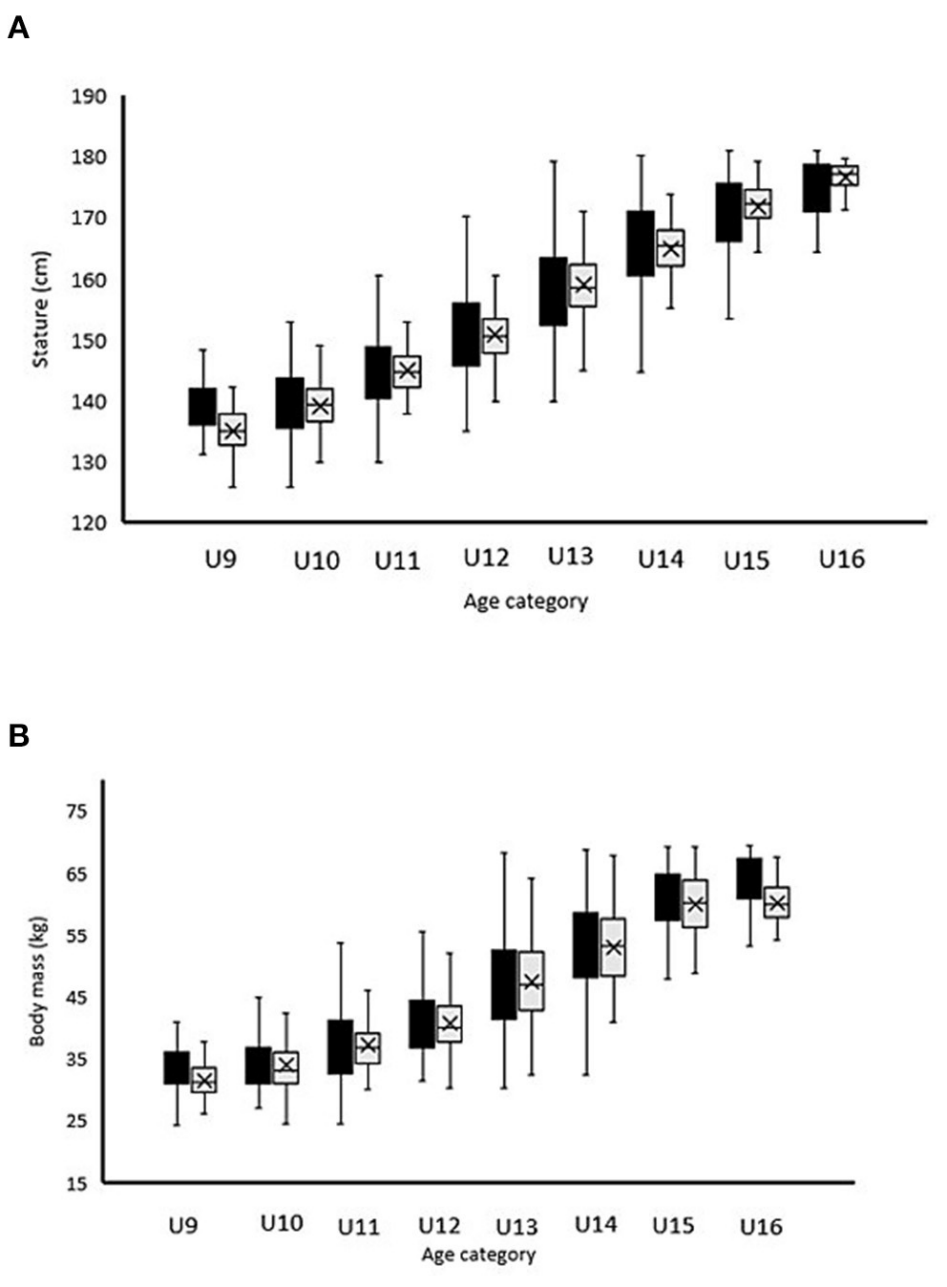
Mean ± SD for anthropometric [**(A)** stature; **(B)** body mass] characteristics of 812 UK academic soccer players according to the traditional categorization based on chronological age (black bars) vs. the newly proposed reallocation method (gray bars).

In Table 6, the mean ± standard deviation (SD) with associated effect sizes and coefficient of variation for anthropometric and physical parameters according to both chronological age and reallocation age are listed. For the anthropometric data, mean CV% of stature (−2%) and body mass (−3.5%) both reduced following reallocations. The U10 squad demonstrated the largest reduction in CV% of the anthropometric characteristics after allocation (stature: 3.6 and body mass 7.6%) (Figure 6).

**Figure 7:**
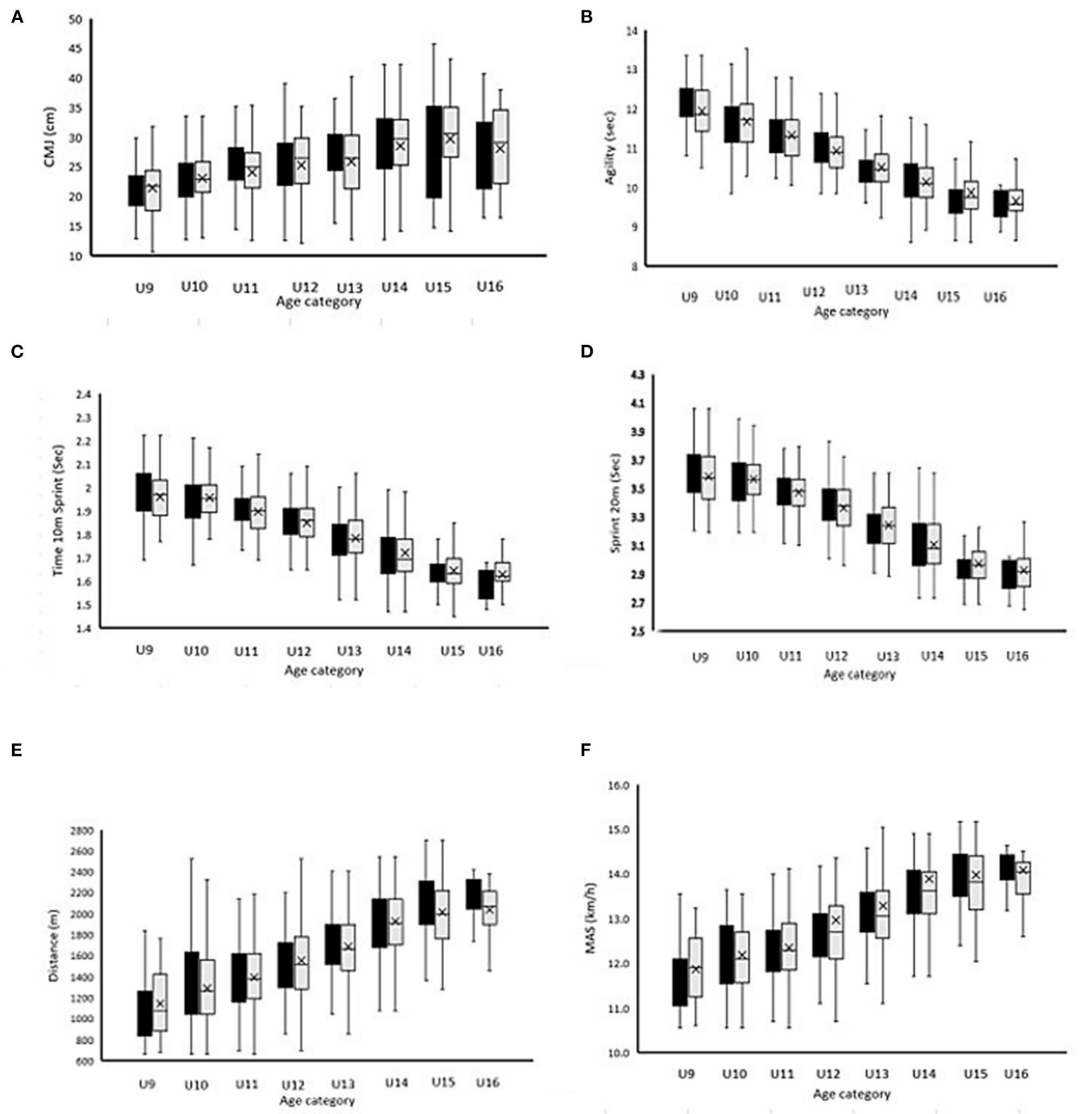
Mean ± SD for physical [**(A)** CMJ; **(B)** Agility; **(C)** 10 m sprint; **(D)** 20 m sprint; **(E)** Distance MSFT; **(F)** MAS MSFT] characteristics of 812 UK academic soccer players according to the traditional categorization based on chronological age (black bars) vs. the newly proposed reallocation method (gray bars). CMJ, counter movement jump; MSFT, multi-stage fitness test; MAS, maximal aerobic speed.

After comparing the eight anthropometric and physical parameters for all squads following reallocation, 45.3% of the comparisons present reductions in CV% (Table 6). By exploring the impact of reallocation, comparing the 6 physical parameters for all squads, CV% was reduced by 27.1% of the metrics, similar to 33.3% of the metrics, and increased by 39.6% of the metrics (Table 6). The U10 and U14 squad demonstrated the greatest reductions in CV% for the physical characteristics (Figure 7; Table 6). The U10 squad showed a reduction in CV% for physical parameters such as agility, 10 m sprint, distance, and MAS in the MSFT. The U14 squad showed a reduction in CV% CMJ (0.3%), distance (0.3%), and MAS (1.4%). Following reallocation, the U9 squad demonstrated a reduction in CV% for agility (0.8%) and MAS (0.1%) during the MSFT. After reallocation, the physical parameters in the U11 and U13 age categories demonstrated no reduction in CV% (Figure 7; Table 6). The CMJ presented a reduction in CV% in the older age categories as U12, U14, U15, and U16 following reallocation. In the same way, physical performance outcomes such as agility demonstrated a reduction in CV% after reallocation in the younger age categories U9, U10, and U14. The CV% for sprint capacity (10 m and 20 m) was only marginally (reduction CV% <0.1%) impacted after reallocation. Especially in the older age categories, reductions in CV% for sprint performance were observed. Reallocation also presented a clear reduction in CV% for endurance performance outcomes in the younger age categories. The CV% for the distance and MAS of the MSFT (endurance capacity) was reduced in U9, U10, U11, and U14 squads after reallocation (Figure 7). Finally, the greatest absolute reduction in CV% after reallocation was demonstrated in distance and MAS for MSFT (endurance capacity). The older age categories U13, U15, and U16 showed an increase in CV% of physical parameters like agility and distance and MAS for MSFT. On the other side, the youngest age category U9 demonstrated an increase in CV% in CMJ and sprint capacity.

## Discussion

The primary aims of the study were to examine if the ED categorization method (i) eliminates the RAE, and (ii) reduces maturity-related differences in anthropometric and physical fitness characteristics between players using a broad sample of academy soccer players (under 9–16) from 23 different UK professional soccer academies. The primary findings of the study were three-fold. First, prior to reallocation, there was a clear RAE (OR 4.57). However, such selection bias dissipated (Q1 = 25.3, Q2 = 25.6, Q3 = 22.4, and Q4 = 26.7%) after implementing the newly formed ED birthdate method. Second, following ED birthdate reallocation, the mean within-group difference in stature (16.6 cm) and body mass (6.7 kg) was reduced. However, the mean age difference increased from 1.8 to 3.9 years. Third, 42.7% of the sample would have been reallocated using the ED birthdate method into a different age grouping compared to the current traditional methods for categorizing players.

Selection is not only associated with receiving better coaching and facing better opponents (Helsen et al., 1998); being involved in higher standard competition levels is more prestigious and, therefore, likely to increase one's motivation and self-esteem (Helsen et al., 1998; Musch and Grondin, 2001). A growing number of publications suggest that a mixture of physical, cognitive, emotional, and motivational factors work together to produce RAEs (Cobley et al., 2009; Doncaster et al., 2020; Romann et al., 2020b; Towlson et al., 2021b) and maturity selection bias. All sport governing bodies are committed to providing equal chances for sports participation and success (Musch and Grondin, 2001; Cobley et al., 2009). It is very likely that many promising players have been overlooked in the past because they suffered from a relative age disadvantage in their early childhood (Helsen and Starkes, 2020). Furthermore, from a public health perspective, physical activity in children and adolescents is considered very important for disease prevention and health promotion (Musch and Grondin, 2001). The long-term result of RAEs will lead to a decrease in the overall quality of the highest competitive teams because players with proper technical potential are overlooked at an early age due to a lack of physical development that is simply related to the period of the selection year in which they were born (Helsen et al., 2005; Jackson and Comber, 2020). For all these reasons, coaches and practitioners must fully understand the potential effects of relative age differences within chronologically age categorized formats. Efforts to recognize valuable remedies to the relative age (and maturity selection bias) problem should be intensified (Roberts et al., 2020). Although the RAE has been well established, the awareness of coaches and institutions seems to be inadequate to reduce the selection bias (Towlson et al., 2019). In their interactions with relatively younger players, coaches should always concentrate on the player's improvement rather than conducting unfair comparisons with a player's relatively older peer (often due to a focus on winning).

Although we acknowledge that relative age and maturation should be considered as separate constructs, a recently proposed solution to the latter is bio-banding (Cumming et al., 2018; Abbott et al., 2019; Bradley et al., 2019; Malina et al., 2019; Romann et al., 2020a; Towlson et al., 2021a). Instead of the typical age-grouping system, young players are divided into teams based on their estimated maturity status using estimate equations based on normal growth curves, child anthropometric characteristics, and/or mid-parent height (Khamis and Roche, 1994; Mirwald et al., 2002; Moore et al., 2015; Fransen et al., 2018; Kozieł and Malina, 2018). Bio-banding is an effective method for reducing the variance in anthropometric characteristics (Macmaster, 2021), but it may increase the variance in age and may be difficult to implement due to the limited number of players per chronological age group (Towlson et al., 2021a). Although bio-banding provides a more equitable playing environment characterized by establishing anthropometrically homogenous groups, it may cause organizational problems because youth players from up to 5 different age categories are merged.

A newly suggested solution for the RAE is the division of youth players by allocation date (Helsen et al., 2021). This allocation date is the midway point between the chronological and the developmental birthdate. Reallocating players by the allocation date leads to a reduced variance in physical characteristics (Table 6) but also limits the variance in age differences, compared to bio-banding (Helsen et al., 2021).

The Union of European Football Associations (UEFA) Financial Fair Play regulation states that all European professional soccer clubs are to operate within their financial means to avoid any financial or competition penalties (Müller et al., 2012). In an attempt to promote “home-grown” talent to compete at the senior level and reduce club financial commitments to imported players, there is a need for professional soccer clubs to install ongoing talent (de)selection strategies that are free from (sub)conscious, transient, maturity-related selection bias (Helsen et al., 1998; Lovell et al., 2015; Towlson et al., 2017). The over-selection of academy players who are either relatively older (Helsen et al., 1998, 2012) and/or possess enhanced maturity-related physical characteristics is well-documented (Lovell et al., 2015; Towlson et al., 2017). Unfortunately, few studies have examined the effect of recently proposed growth and maturation reallocation methods such as on match-play characteristics (Lüdin et al., 2021; Macmaster, 2021) or offered pragmatic solutions to reduce the over-selection of early-maturing players (Helsen et al., 2021).

Despite the over-selection of both relatively older and early maturing (Helsen et al., 1998, 2012), taller players for soccer development programs (Lovell et al., 2015; Towlson et al., 2017), a multi-disciplinary approach to understanding the effects of “re-categorizing” players according to their estimated development age during ongoing talent (de)selection is of particular relevance to academies and governing bodies across the world.

The conclusion of the first part of our study can be summarized as follows: (1) The age distribution for chronological age per quartile demonstrated a clear RAE (Figure 3). RAEs have been clearly found for the whole database and every individual age category, except the U9 (Figures 1, 3); (2) Using the new allocation method, 42.7% (*n* = 347) of players would be allocated to a different squad compared with distribution by chronological age. Specifically, 17.7% would be reallocated 1 squad lower, 20.1% 1 squad higher and 3% would be advised to be reallocated 2 squads higher. Less than 1% would be reallocated 3 squads lower (Figure 5; Table 5); (3) 29 of the 64 (45.3%) observed between-method comparisons for anthropometric and physical characteristics demonstrated reduced group CV% (Figures 6, 7; Table 6); (4) Reductions in group CV% for specific age categories and physical characteristics could be partially related with maturity (peak height velocity). However, RAE is a multifactorial construct that needs to be assessed independently (Towlson et al., 2021b). Because of the two-part design of the study, these findings will be separately evaluated, starting with phase 1.

### Phase I: Reallocating Youth Players Using the Midway Point Between the Chronological and Estimated Developmental Birthdates

In this first part of the study, our aim was to quantify the presence and magnitude of the relative age effect in each annual age category spanning the entire cycle of player development at the representative level in English academy youth soccer.

Using the traditional age-based categorization, the relative age effect (RAE) is clearly present for the whole database and each age category (Figures 1, 3). Relative age distributions between quartiles for each age category are shown in Figure 1.

Regarding the first finding, the magnitude of the RAE was a factor of “five” (4.57) for the overall sample of youth players (Q1 = 45.0 vs. Q4 = 9.8%) (Figure 3). An uneven distribution was identified for each age group, with 37.5–52.2% of players born in Q1 and 6.3-13.3% in Q4 (Figure 1). Specifically, this means that a five-fold overrepresentation was consistently found for young players born in the first quartile of the selection year (meaning the first three months after an age-grouping cut-off-date of the first of September) as well as an underrepresentation of players born in the last quartile (the last 3 months). Likewise, for the entire cohort (i.e., across U9-U16's), there was a 4.57 greater chance of being enrolled into a player development program if you were relatively older (Q1) vs. being relatively younger (Q4). A clear over-representation of Q1 players was observed at U10 (OR: 6) and progressively decreased until U12 (OR: 2.82). Thereafter, ORs were still high between U13-U16 (5.11–6.86). This typical RAE was also reported in former studies (Figure 1) (Lovell et al., 2015). These findings illustrate that relatively older players (quartile 1) of their selection year were 4.57 times more likely to be registered and participate in development academic programs compared to the quartile four (relatively younger) players, with the bias most pronounced at U10 and U16. The Odds ratios of RAE were comparable with a broader study of English young players (Lovell et al., 2015) but larger than those determined in Belgian soccer academies (OR's: 0.7–3.6) (Helsen et al., 2021). This suggests that a substantially large RAE is present within the UK youth academies. These data reflect the inherent broader regional, cultural, and racial characteristics, including the diversity of playing styles, influences, and recruitment philosophies in soccer academies (Steingröver et al., 2017).

RAEs were clear and obvious at the start of the talent development programs (U9, U10), with Q4 players 5.34–6 times less likely to be registered and participate in competitions and training programs compared to Q1 born players (Figure 1). The substantial RAE in U9, U10 and U15, U16 squads in our dataset suggests that the anthropometric and physical developmental differences between the relatively older and younger players are most pronounced at this stage. The large RAE in the U16 squad may probably be biased by the small sample size of 14 players. With respect to physical fitness, relatively older players from the U9, U10 squads demonstrated superior agility and endurance performances (Table 6). The anthropometric and fitness differences between Q1 and Q4 might again be partially explained by the more advanced growth status and younger APHV of selected Q4 players (Lovell et al., 2015). However, as Hill et al. (2020) have shown, relatively younger players may also be characterized as possessing advanced maturation characteristics. Therefore, maturity-specific explanations are difficult to confirm. Finally, this suggests that players at these ages demonstrated an equal anthropometric and fitness phenotype, whereby relatively younger players with advanced normative growth and maturation, were selected to receive advanced coaching opportunities in training and competition.

In the U11, U12, a clear and obvious decline in the RAE magnitude (2.8–3.6%) was observed (Figure 1). A small increase in the number of Q4 players (12.2–13.3%) was also noticed. This could be partially explained by the fact that the normative growth declines to its lowest rate since birth at ~11 years of age, after which a rapid acceleration toward peak height velocity from circa-12 years begins (Philippaerts et al., 2006; Lovell et al., 2015). The relatively older players are advantaged by an earlier onset of the adolescent growth spurt. A selection bias toward players advanced in maturity status for chronological age emerged in U12 players and increased with age. Professional football academies need to recognize relative age and maturation as independent constructs that exist and operate independently (Hill et al., 2020). Relatively older players in these squads also possess small physical advantages in terms of sprint and agility performance, as evidenced by the reduction in CV% for these physical parameters after allocation (Figure 7; Table 6). This can be an important underlying factor in the RAE given the importance of speed performances in talent selection outcomes and goal situations.

In the U13 and U14, larger magnitudes of anthropometric advantages for Q1's were observed in combination with the faster approach to peak height velocity during maturation (Table 1) (Towlson et al., 2017). This faster approach to peak height velocity for the relatively older players during maturation at the onset of pubescent growth spurt could partially contribute to the increasing RAEs observed from this point onward until U16 (Figure 1). Physical characteristics showed small speed (10 and 20 m sprint) and power (CMJ) advantages by reduced CV% after allocation (Figure 7; Table 6). These physical advantages afforded to relatively older players may partially contribute to RAE during the pre to circa-PHV transition, although RAE is a multifactorial event (Towlson et al., 2021b).

The U16 RAEs remained high (OR: 5.9–6.9%). After reallocation, a reduction in CV% in anthropometric and physical characteristics like sprint performance and CMJ performance was observed, although the U16 squad was biased because of the small sample size for this age category (Figure 7; Table 6). RAE could partially be explained because of the “cascade” effect and the difference of anthropometric and physical advantages from earlier adolescent growth spurts that were observed in prior age categories (Lovell et al., 2015).

In conclusion, the RAE magnitude (OR) tends to decrease with increasing chronological age. The largest discrepancies in quartile proportions have been associated with young age-cohorts (U9–U10) (Figure 1). At the pre-adolescent stage, where the peak height velocity in maturation declines to its lowest rate since birth in combination with the largest reduction in CV% in anthropometric characteristics (Figure 6; Table 6) (Philippaerts et al., 2006; Lovell et al., 2015; Towlson et al., 2018), the role of RAE in talent selection may be most influential. Thereafter, normative growth effects are partially biased/compensated by the influence of biological maturation. Relative age and maturity clearly confound the physical and talent development processes; however, these effects operate independently of one another (Towlson et al., 2021b).

Before reallocation, the mean differences in player stature and body mass by chronological age distribution were 40.3 cm and 33.2 kg, respectively (Table 1). After reallocation, these differences were clearly reduced to 23.7 cm and 26.5 kg, respectively, possibly due to players being more closely matched to maturation status (Table 2). The mean age difference increased from 1.8 years to 3.9 years (Tables 1, 2). The largest reductions in mean differences of anthropometric parameters like stature were observed in the U9 and U14 age categories (Figure 6; Table 6). As stated before, the U14's larger magnitudes of anthropometric advantages for Q1's were observed in combination with the faster approach to peak height velocity during maturation and were partially linked with a larger RAE in these age categories (Philippaerts et al., 2006; Lovell et al., 2015). Reductions in mean differences (delta values and CV%) of the anthropometric parameters were clearly observed in each age category (Figure 6; Table 6). For more than half of the players (57.3%, *n* = 465), reallocation to another age category was not recommended (Figure 5; Table 5). A total of 42.7% of the players of the current database were reallocated to another age category. These observations are similar to the first reallocation study of Helsen et al. (2021). Reallocation percentage per age category is almost the same with small, enhanced values for the younger (U9, U10) and older age categories (U15, U16) and smaller percentages for U11 and U13 (Table 5). For the younger age categories (U9, U10), this can be partially linked to the larger RAE (as previously mentioned) and also to more enhanced differences in maturity pre-PHV (Lovell et al., 2015). In this respect, professional football academies need to recognize relative age and maturation as independent constructs that exist and operate independently (Hill et al., 2020). The higher reallocation percentages for the older age categories can be explained by the higher RAE and the cascade-effect. Reallocation data in the U16 are biased because of the small sample size (*n* = 14).

Small reallocation degrees in the U11 (Table 5) can also partially be linked to the smaller RAE in this squad because of the lowest normative maturity growth speed at approximately 11 years of age (Lovell et al., 2015).

While the difference in stature and body mass is clearly reduced in the reallocation group (Table 2; Figure 6), they are more likely to develop adaptive learning skills and better technical and creative skills. Further research is necessary to examine if these adaptive learning skills can become an additional advantage in adulthood when physical differences have attenuated or even reversed. In addition, other issues that are linked to reallocating players to higher or lower age categories need to be considered, e.g., social and psychological factors (Towlson et al., 2021a). That is why a multi-disciplinary approach to understanding the effects of “re-categorizing” players according to their estimated development age during ongoing talent (de)selection is of particular relevance to academies and governing bodies across the world (Lovell et al., 2015; Towlson et al., 2019). Previous studies reported that academy recruitment staff place greater value on psychological characteristics than technical/tactical, and physical factors during the talent selection (Towlson et al., 2019). Specifically, recruitment staff value psychological factors more than medical, sport science, and fitness staff (Towlson et al., 2019). Similarly, they also value psychological factors more than medical staff in the evaluation of player maturity (Towlson et al., 2019). Efficient respectful communication between the coaching staff, the player, and his/her parents about the importance of this reallocation for appropriate long-term player development is certainly recommended, especially if players are reallocated to lower age categories to prevent further drop-out (Helsen et al., 2021).

### Phase II: Evaluation of the Within-Group Variation of Anthropometric and Physical Fitness Characteristics Based on Chronological and Estimated Developmental Birthdates

The present study provided promising evidence that the newly proposed player (re)allocation method is an appropriate strategy for reducing transient, maturity-related anthropometric (physical fitness to a lesser extent) characteristics (Figure 6; Table 6), which are often afforded to early maturing players (Helsen et al., 2021). The reallocation method has the potential to remove the relative age bias (Figure 3), with 45.3% (29/64 comparisons) of the observed between-method comparisons showing that the reallocation method reduced the group coefficient of variance for maturity, physical (Figure 7), and anthropometric (Figure 6) player characteristics. The reallocation method reduced clearly group CV% for anthropometric characteristics for all of the sampled squads (Figure 6; Table 6).

After reallocation, 27.1% (13/48 comparisons) of the group CV% for physical characteristics was reduced (Table 6). U10 (75) and U14 (62.5%) were the age categories where group CV% for most physical fitness characteristics were reduced after allocation (Table 6; Figure 7). In the U11 (0%) and U13 (0%) group CV% for the least parameters of physical fitness was reduced (Table 6). By analyzing this data of the reallocation method in more detail, the relationship between relative age, maturation, anthropometry, and physical fitness characteristics could be partially assessed. However, maturation and relative age should be considered as independent entities because the interpretation of the influence of maturation and relative age on player development programs is constantly evolving (Towlson et al., 2021b).

The substantial reduction of group CV% in the U10 after allocation (Table 6) can be explained because of a large RAE (Figure 1) in this age category. The younger age categories U9 and U10 showed a reduction in group CV% for physical fitness parameters such as agility and endurance (distance MSFT and MAS MSFT) and an increase in group CV% for physical parameters as CMJ and sprint capacity (Figure 7; Table 6). The high RAE in the U9 and U10 suggests that the anthropometric and physical developmental differences between the relatively older and younger are most pronounced at this stage at the pre-PHV. In terms of physical fitness, relatively older players at this stage did demonstrate superior agility performance and endurance parameters (Table 6; Figure 7). With respect to development, previous studies showed that pre-PHV soccer players showed superior aerobic running economy compared to circa-PHV players (Philippaerts et al., 2006; Lovell et al., 2015; Towlson et al., 2018). Group CV% for 10 m sprint performance was also reduced in the U10 squad (Table 6; Figure 7). Previous studies conducted in male youth players revealed that speed training demonstrated greater adaptive responses in circa-PHV and post-PHV groups compared to pre-PHV groups (Philippaerts et al., 2006; Lovell et al., 2015; Loturco et al., 2020). The smallest reduction in group CV% of physical fitness parameters in the U11 can partially be explained by the decline in RAE and the lowest normative growth velocity since birth. Small reductions in group CV% were still observed in physical fitness parameters as agility and endurance (Table 6; Figure 7). Superior agility performances were also observed in this age category in previous studies (Lovell et al., 2015; Towlson et al., 2017). Training experience (determined by years of training) was more closely associated with aerobic performance rather than with maturity *per se*, as already mentioned in previous studies (Lovell et al., 2015). This can further illustrate the improvement in aerobic performance in the U11 squad.

In the U12–U14 squads, we noted a reduction in group CV% in physical fitness parameters as jump and speed performance (CMJ, sprint 10/20 m) (Table 6; Figure 7), which coincided with the approach to peak height velocity during maturation. These distinct advantages for the relatively older players during the onset of the pubescent growth spurt are also likely to contribute to the increasing RAEs observed from this point onward until U16 (Figure 1). The precise underlying mechanism for greater sprint and jump capacity at this stage may relate to a range of biological, neurological, and biomechanical factors that interplay during maturation (Lovell et al., 2015; Towlson et al., 2017, 2018; Loturco et al., 2020). Because of the greater stature and body mass of relatively older players, we speculate that increased muscle length and cross-sectional area could be the cause. Circa-PHV larger speed and power performances were observed with a reduction of CV% after allocation (Table 6, Figure 7), which can be partially related to the RAE during this period. Previous studies showed that age-related enhancements in sprint running performances were almost exclusively related to differences in maturation rather than anthropometric factors *per se* (Lovell et al., 2015; Towlson et al., 2018; Loturco et al., 2020). These players may benefit from enhanced neural function, coordination, muscle architecture, and hormonal-induced increases in muscle power. This may be an important factor in the RAE and allocation method, given the value of speed qualities in talent selection outcomes and goal situations.

In the older age categories, U15 and U16, further reductions in group CV% were observed after allocation (Table 6; Figure 7). This is in line with the large RAEs in these age categories and can be indicative of a “cascade” effect. Previous studies showed that speed training demonstrates greater adaptive responses in circa-PHV and post-PHV groups compared to pre-PHV groups which will cumulate at the U16 because of the cascade-effect (Lovell et al., 2015; Towlson et al., 2018). Additionally, growth-related musculoskeletal adaptations like tendon, fascicle length, and motor unit recruitment patterns, settle post-PHV and better represent adult characteristics, predisposing athletes to both an increased magnitude and rate of force- and speed-development potential (Lovell et al., 2015; Towlson et al., 2018).

The increase in CV% in specific physical fitness parameters after allocation, which is observed in some age categories (Table 6; Figure 7), can partially be explained by the mixture of ethnicities in some playing groups in the UK and the fact that some physical characteristics like sprint and jump capacity are multifactorial. This means they are not only based on absolute anthropometric values, but also on maturity, experience, and other factors. However, it should be acknowledged that the determinants and requirements of the RAE are non-linear and multifactorial (Towlson et al., 2021b). The RAE is reinforced in teams with a larger variety of nationalities or ethnic groups (Steingröver et al., 2017). In that way, the RAE is more pronounced in teams from urban areas, compared with those from rural areas. In conclusion, RAE is influenced by multiple factors as maturity, experience, ethnicity, personal development, psychological, and social factors (Towlson et al., 2021b).

Teunissen et al. (2020) confirmed that none of the four evaluated prediction equations is accurate for estimating APHV in individual players nor are predictions stable over time, which limits their utility for adjusting training programs. As such, this new reallocation method provides an efficient solution to address relative age- and maturity-related bias in soccer. These findings are of practical relevance and importance for coaches given that maturity and relative age selection bias can partially contribute to the premature deselection, playing position allocation of academy soccer players, and early drop-out of players. This can confound the (de)selection processes of academic soccer development centers across the world and likely limits the size of the talent pool for clubs and nations to select from. Therefore, this study provides persuasive evidence for the application of a new player allocation method to remove the temporary, physical fitness, and anthropometric advantages afforded to older and more mature players. In addition, this study provides clear evidence that this new method for categorizing players removes the RAE (Figures 3, 4) and creates a more “level playing field” by reducing the within-group variation of somatic and physical fitness characteristics (Table 6; Figures 6, 7). This provides relatively smaller players the opportunity to develop their talents fairly.

## Limitations Of The New Reallocation Method

Although this study demonstrates early promise, there are still a few limitations. First of all, the ED birthdate was calculated using a developmental age calculator which is based on normative Belgian growth curves (Roelants et al., 2009). Therefore, the curves used in this study as a reference point for UK youth players are potentially limited as they are only based on the growth data of Belgian children. The stature and body mass of Belgian youth players are comparable with other youth players in Europe, but growth curves that are specific for every country may have a slightly different impact on the estimations. Another consideration is that this study implemented a specific target group of academy youth soccer players. Depending on the geographical area, some clubs in the UK may involve particular differences in ethnicity (Steingröver et al., 2017). In contrast, the growth curves used here were based on a broader Belgian population of mixed ethnicity. This issue could be solved by creating specific growth curves by nationality for youth players, eventually even by sport with estimated differences in ethnicities. Another limitation is that the CMJ test can be difficult to perform for younger soccer players (< U14) because of the lack of enhanced neural function, coordination and fascicle length, and motor unit recruitment patterns. This may create bias in the interpretation of results with respect to the jump quality.

Also, despite the fact that this new reallocation method eliminated the RAE (Figures 3, 4) and reduced the between-group variation in maturation status (Table 6; Figures 6, 7), the effect on psychological, cognitive, and social parameters in a training and match context have not yet been shown.

## Future Directions For Research

According to the literature, RAE is determined by different players' anthropometric dimensions, which could be highly considered in the most competitive sports environment, such as soccer (Brustio et al., 2018; Lupo et al., 2019). Therefore, the results of the present can be emphasized as a key explanation and contribution to better understand the RAE. Further investigations using this newly proposed player allocation method are required to better understand the overall impact of our method on the temporary, age-, and maturity-related differences between players. Therefore, coaches, clubs, academies, and governing bodies should take a coordinated approach to player development research and work collaboratively to aggregate players' relative age, maturity, physical fitness, development, and anthropometric datasets to truly understand the impact of both relative age and maturity selection bias in training and competition on physical, psychological, cognitive, technical, and tactical characteristics. This can offer further insight into the effectiveness of new approaches to further remove such selection bias. Also, the impact on psychological and cognitive parameters needs to be evaluated, as psychological factors are considered a priority by practitioners during the talent selection process (Towlson et al., 2021a).

The speculation that the reallocation of youth players will lead to lower drop-out, more efficient talent selection, more specific training and match loads, and fewer injuries need to be assessed in reallocated groups.

The reallocation method uses the midway point between the chronological and ED birthdates. Perhaps more weight should be given to either the chronological or developmental birthdates. Alternatively, it would also be interesting to examine to what extent the inclusion of weight data in addition to stature data may have an impact on the reallocation figures. Evidently, in future studies, we may address female soccer players as they also have substantial drop-out (Møllerløkken et al., 2015). Social pressures that encourage adolescents to conform to gender-based stereotypes could prevent females from achieving excellence in competitive soccer, especially early-maturing females (Vincent and Glamser, 2006). The physical characteristics needed for athletic accomplishment are sometimes opposite to the representation of the ideal female body. Thus, such role conflicts could lead outstanding female players to drop out from soccer. If early physical development acts as an important advantage for young males in many sports, it may also act as a socially constructed disadvantage for young females which could escalate their drop-out from sports activities. Moreover, the current analysis provides useful information on the potential physical performance improvement for young soccer players with age. Appropriate magnitudes of improvement in physical performance capabilities have also been provided to help determine normal changes over a player's career or a shift in training emphasis by coaches and scouts. Although the development of physical performance is a crucial element of soccer preparation and talent identification, all other areas of performance (technical, tactical, psychological, or cultural) should be considered as objective selection criteria to properly evaluate a player's quality and to improve talent identification. Finally, this study provides a static snapshot of RAE across ages at one point in time. Maturation, RAE, talent selection, and development are non-linear independent dynamic processes (Towlson et al., 2021b). So, influences on athlete success, development, and drop-out for each age category in the long-term need to be processed. However, this new allocation method shows early promise in the goal of “leveling the playing field.”

## Practical Implications

The reallocation method may offer a useful tool for further evaluation of the successful ongoing (de)selection of talented young soccer players according to anthropometric, physical, and psychological determinants of training involvement and match performance and will help practitioners make more and better-informed decisions regarding their choice of method for matching players. Concerning the implementation of this new allocation method, the most efficient period to evaluate anthropometric and physical fitness may be toward the end of the season to give clubs and national associations proper time to compose the youth categories for the next season. One measurement per season before a growth spurt and two measurements during growth spurt around PHV of maturation would be appropriate to avoid a reorganization in-season. Coaches should ensure that they use high-quality standardized equipment for their measurements, with consistent protocols and procedures to ensure measurement validity. As a result of reallocation, players will be more challenged to develop technical and tactical skills because the differences in player stature and body mass per age group are equalized (Abbott et al., 2019). Being dropped to a younger age category may influence the social relationships of players, as they are no longer competing in the same team as their social peers. There is also a need for sports scientists to educate key stakeholders, such as coaching staff and parents, about maturation and RAE, particularly in relation to the potential use of the allocation method within their player-development strategies.

Furthermore, it is recognized that academy practitioners are challenged by the complexities associated with prescribing suitable long-term athlete-development plans to players and parents to engage a multi-disciplinary approach with appropriate training to develop further technical, physical, and psychological characteristics safely and efficiently (Towlson et al., 2019). The imbalance between strength and flexibility may partly explain maturity-injury associations. The adolescent growth changes result in increased demands being placed on muscular, tendinous, and ligamentous structures during biological maturation when they are exposed to repeated high competition and training loads. Prescribing appropriate training loads for particular maturity-based age categories after allocation may reduce injury risk to a minimum. Development-based categorization methods also provide more opportunities for youth players to engage key psychological constructs, which are important when assessing talent (Cumming et al., 2018; Towlson et al., 2019). Finally, it is essential to establish clear lines of communication between players, parents, and coaching staff about the purpose of the format of reallocation to improve the long-term development of the individual players and to reduce drop-out (Cumming et al., 2018; Towlson et al., 2019). In conclusion, reallocation of young players as suggested here may result in less drop-out, more efficient talent selection, more specific training and match loads, and thus fewer injuries.

Coaches can potentially up- or downshift the athlete development programs according to age categories based on periods of accelerated gains in relation to PHV to make them more efficient. Training to improve physical fitness qualities in young soccer players is a longitudinal process that involves the systematic manipulation of training load, incorporating the different aspects of the demands of match play. Therefore, the consideration and specific implementation of maturation within the long-term athlete development model for youth soccer is very important for coaches.

This data can inspire the improvement of the development of targeted injury-prevention programs for young players (FIFA 11+) to improve functional age-specific performance and enhance physical characteristics, like dynamic balance and agility. Finally, our findings can be used to inform the national governing body about talent identification and medical processes for specific injury prevention, while providing educational resources and courses. Instead of leaving the initiative to individual clubs, it is much more efficient to organize a structural European or global solution that is implemented by the national governing bodies for a given level of competition where teams are involved.

## Conclusion

In summary, the findings of our study demonstrate that this innovative reallocation method provides an appropriate tool for the successful ongoing (de)selection of talented young soccer players according to anthropometric and physical determinants of training and match-play performance. It is cost-effective and easy to implement in existing organizational structures of youth football for clubs and associations *via* the newly developed developmental age calculator. By using an extensive dataset of 1,003 players of multiple youth football teams in the UK, the findings demonstrated, first of all, that this new method would go a long way to “level the playing field” with respect to stature, body mass differences, and physical fitness and also result in a more even distribution of birthdates throughout a selection year. In addition, reallocation reduced the between-player variation in maturity, anthropometric, and physical characteristics when compared to “traditional” chronologically categorized youth groups. The current study permits a better understanding of the effect of development-based categorization of players on the anthropometric, physical, and psychological aspects of young players during training and match-play and helps coaches make more informed decisions regarding their choice of method for matching players. The reduction of relative age- and maturity-related bias leads to fewer drop-outs and thus a larger player talent pool for selection. Finally, from a public health perspective, physical activity and mental health in children and adolescents are considered very important for disease prevention and health promotion. As such, the retention in sports of as many youth players as possible, for as long as possible, and in the best learning environment possible to develop technical and tactical skills would be the aim to improve general population health. So, these important values need to be adopted and reached by national soccer associations and domestic (professional) soccer clubs by installing ongoing talent (de)selection strategies that are free from (sub)conscious, transient, maturity-related selection bias.

## Data Availability Statement

The raw data supporting the conclusions of this article will be made available by the authors, without undue reservation.

## Ethics Statement

The studies involving human participants were reviewed and approved by Research Ethics Committee KU Leuven, Belgium. Written informed consent to participate in this study was provided by the participants' legal guardian/next of kin.

## Author Contributions

WH initiated, supervised the research from the very beginning, published this new method in a first research article about leveling the playing field in the same journal as this study (Helsen et al., 2021), and also developed the online calculator that can be used by national associations and clubs to reallocate their youth players to a higher or lower team that is more in line with their physical development. SB contributed to the study in the context of her master's thesis and also performed the analytic calculations, numerical simulations, and drafted the first versions of the manuscript. CT was also involved from the start, contributing to all stages of the study, the interpretation of the results and was assisted by GP, in particular, for the data collection. WH, JS, CB, and CT revised the preliminary drafts as well as the final version. All authors provided critical feedback and fine-tuned the research, analysis, and manuscript.

## Conflict of Interest

CB was employed by CB Sports Performance Ltd. GP was employed by company Pro Football Support. The remaining authors declare that the research was conducted in the absence of any commercial or financial relationships that could be construed as a potential conflict of interest.

## Publisher's Note

All claims expressed in this article are solely those of the authors and do not necessarily represent those of their affiliated organizations, or those of the publisher, the editors and the reviewers. Any product that may be evaluated in this article, or claim that may be made by its manufacturer, is not guaranteed or endorsed by the publisher.
